# Efficacy of the Combination of rhBMP-2 with Bone Marrow Aspirate Concentrate in Mandibular Defect Reconstruction after a Pindborg Tumor Resection

**DOI:** 10.1155/2020/8281741

**Published:** 2020-03-18

**Authors:** Kamel Alraei, Jameel Sharqawi, Somaya Harcher, Ibrahim Ghita

**Affiliations:** ^1^Department of Oral and Maxillofacial Surgery, Al-Noor Specialist Hospital, Makkah, Saudi Arabia; ^2^Department of Oral and Maxillofacial Surgery, Royal Commission Hospital, Jubail, Saudi Arabia; ^3^General Dentist, Saudi Arabia; ^4^Department of Hematology, Al-Noor Specialist Hospital, Makkah, Saudi Arabia

## Abstract

Recombinant human bone morphogenetic protein-2 (rhBMP-2) is an osteoinductive growth factor used in oral and maxillofacial surgery. It offers a feasible alternative for various regenerative procedures, including reconstruction of mandibular defects. In this study, we report a case of a large Pindborg tumor involving the left mandible. The treatment consisted of surgical resection, followed by off-label use of rhBMP-2 in addition to bone marrow aspirate concentrate, together with an allograft in a titanium mesh. The patient was rehabilitated with dental implants, and a good clinical outcome was achieved. We found no evidence of bone resorption or complications in both clinical and radiographic evaluations during the one-year follow-up period. In conclusion, we have demonstrated the efficacy of using rhBMP-2 combined with bone marrow aspirate concentrate, and an allograft with a titanium mesh, for the reconstruction of long mandibular bone defects. Not only is this combination feasible, but it also has the advantages of lower morbidity and cost.

## 1. Introduction

The reconstruction of mandibular defects poses a significant challenge in oral and maxillofacial surgery, particularly in cases with bone continuity defects [1]. Mandibular defects can be a result of trauma, benign tumors, or malignancies [2]. For successful reconstruction of mandibular defects, the surgeon must establish mandibular continuity, in addition to restoring the height and width of the bone to allow further dental rehabilitation [3].

Autogenous bone grafts are considered the gold standard for surgical repair of most osseous defects [4, 5]; however, there are major drawbacks to this treatment modality, including donor site morbidity and the limited amount of bone that can be harvested [6, 7]. With recent breakthroughs in tissue engineering, many new graft materials and treatment modalities for the reconstruction of mandibular defects have been developed. One of the alternatives to traditional bone grafting is the use of recombinant human bone morphogenetic protein-2 (rhBMP-2), which is a potent osteoinductive growth factor that attracts mesenchymal stem cells, and stimulates them to proliferate and differentiate into osteoblasts [8, 9]. rhBMP-2 can be used alone or in combination with different types of grafts, such as allografts and autografts. Allografts are osteoconductive materials, which maintain space for bone ingrowth, whereas bone marrow aspirate concentrate (BMAC) increases the supply of stem cells and cytokines, thus increasing the osteogenic potential of the graft [10].

In this study, we sought to evaluate the results of using rhBMP-2 combined with BMAC, and an allograft with a titanium mesh, for the reconstruction of segmental bone defects.

## 2. Case Presentation

**Figure 1:**
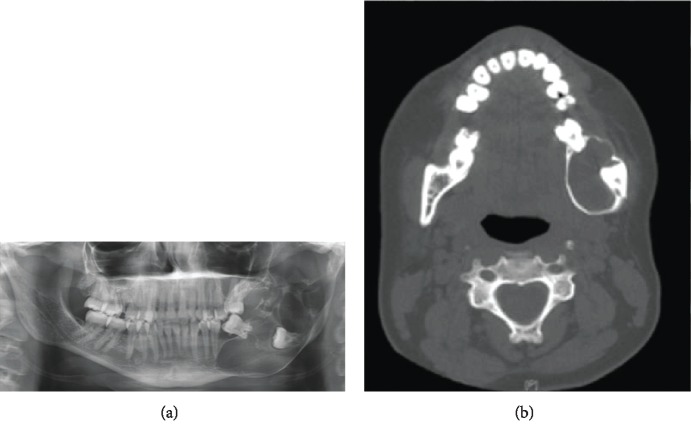
(a) Orthopantomogram showing an impacted 38 with an associated multilocular radiolucent lesion involving the left posterior mandible, which extends from the second premolar to the left condyle. (b) Axial view of a computed tomographic image of the facial bone showing a hypodense lesion in the left posterior mandible, with expansion and thinning of both the buccal and lingual cortices.

**Figure 2:**
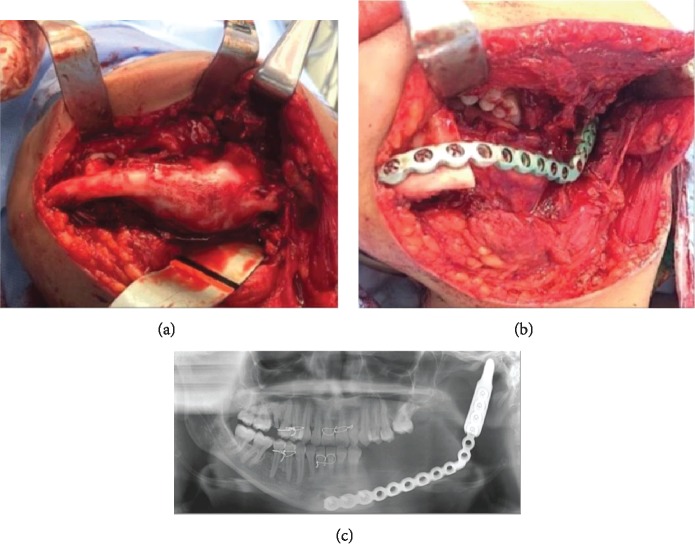
(a) Photograph showing the tumor and the expansion of both the buccal and lingual cortices. (b) Segmental resection of the left mandible stabilized with a 2.7 mm reconstruction plate. (c) Postoperative orthopantomogram.

A 27-year-old woman presented to the clinic for evaluation of a persistent swelling on the left side of the mandible causing facial asymmetry. Radiographic imaging showed a multilocular, radiolucent lesion involving the left posterior mandible, extending into the left condyle (Figure 1). We performed a biopsy which revealed a calcifying, cystic, odontogenic tumor (a Pindborg tumor). As this tumor is aggressive, we decided to proceed with a transcutaneous segmental resection under general anesthesia, followed by immediate reconstruction using a 2.7 mm reconstruction plate (Figure 2). The patient's postoperative course was uneventful. After nine months, the patient returned for the reconstruction of the mandibular defect using rhBMP-2 combined with BMAC, and an allograft with a titanium mesh.

**Figure 3:**
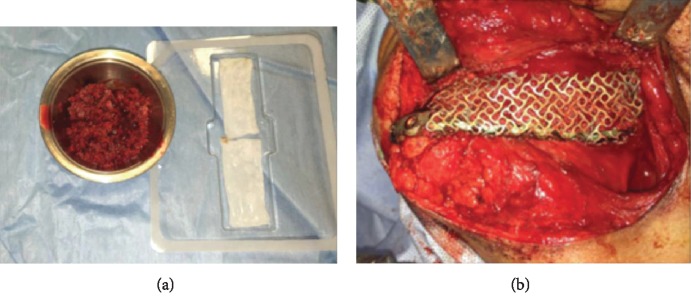
(a) Absorbable collagen sponges (ACS) impregnated with recombinant human bone morphogenetic protein-2 (rhBMP-2) (right side) and allogenic chips soaked in bone marrow aspirate concentrate (BMAC) with small cut pieces of ACS with rhBMP-2 (left side). (b) Photograph showing the entire defect filled with rhBMP-2 combined with demineralized bone allograft and BMAC.

Under general anesthesia, we cannulated the patient's posterior left iliac crest; 60 cc of bone marrow was then aspirated and concentrated using density gradient centrifugation. We placed the patient into maxillomandibular fixation and made a transcutaneous submandibular incision at the scar site of the previous incision. After proper dissection, we exposed the entire defect, together with the reconstruction plate, and recontoured the adjacent bony margins. The titanium mesh was then adapted to reconstruct the mandibular contour, while acting as a protective matrix to contain the graft material. We then secured the mesh in place with a wire. We placed a total of 12 mg of rhBMP-2 (Infuse® Bone Graft from Medtronic Sofamor Danek, Inc.; Tennessee, USA) in four absorbable collagen sponges (1.5 mg/ml). Two sponges were placed inside the mesh, and the two other sponges were cut into small pieces and mixed with 16 mm of allogenic cancellous bone (LifeNet Health; Virginia, USA) in combination with BMAC. The graft material was then packed into the osseous defect. An additional allogenic block (measuring 3 × 1.5 cm) soaked in BMAC was placed in the posterior aspect of the defect, to allow greater dimensional stability (Figure 3). A “watertight” seal closure was performed. The postoperative course was uneventful.

**Figure 4:**
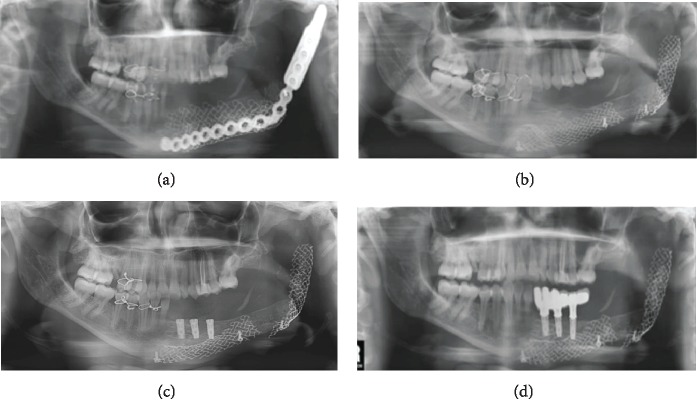
(a) Orthopantomogram (OPG) at the three-month follow-up. (b) OPG after removing the reconstruction plate. Bone regeneration can be observed on the left mandible. (c) OPG showing implant rehabilitation. (d) OPG showing prosthetic rehabilitation with dental implants and an implant-supported partial denture.

The patient showed radiographic evidence of bone formation three months after the operation; mandibular continuity was regained, as demonstrated both radiographically and clinically (Figure 4(a)). After six months of follow-up, the reconstruction plate was removed as it would have blocked the subsequent placement of implants (Figure 4(b)). Three dental implants were placed under local anesthesia (Figure 4(c)), and prosthetic rehabilitation with implant-supported partial dentures was initiated three months later (Figure 4(d)). After four years of follow-up, a good implant stability and cosmetic outcome was achieved.

## 3. Discussion

Successful reconstruction of mandibular continuity defects requires the restoration of both vertical and horizontal dimensions of the bone for further functional rehabilitation and implant placement; this step is essential for the creation of an appropriate facial form [11]. In the case of the patient presented herein, we were able to meet these objectives while minimizing morbidity.

Autogenous bone grafts are still considered the gold standard for the reconstruction of osseous defects since they incorporate all the properties necessary for bone regeneration, i.e. osteogenesis, osteoinduction, and osteoconduction. Furthermore, they are histocompatible, meaning that they do not trigger a specific immune response and are not associated with the risk of infection transmission [12, 13]. The most common donor site for obtaining autogenous bone grafts is the iliac crest; the main advantage of this site is the availability of sufficient quantities of bone with the desired quality [12, 14, 15]. Despite all the benefits, the use of autogenous bone grafts is still associated with a high morbidity and a number of potential complications (namely increased overall time of the surgical procedure, risk of hematoma, pelvic instability, sensory disturbances, poor cosmetic appearance, gait disturbance, infections, herniation of abdominal content, and acute or chronic pain). However, the surgical technique also has an important role in determining postoperative complications [16–18]. The use of autogenous bone grafts in mandibular defects provides a reasonable long-term survival and high success rate (up to 70%); however, the chances of a successful outcome are lower if the defect is longer than 6 cm [19, 20].

Allografts are mainly space-occupying, osteoconductive structures with minimal osteoinductive capability, as they are designed to have minimal antigenicity and risk of infection transmission [21, 22]. Several studies have compared different types of allograft materials in procedures such as ridge augmentation, sinus lift, and bone reconstruction in the maxillofacial region. These studies have concluded that allografts provide an acceptable material for grafting, with the main advantage being the saving of surgical time as no additional bone harvest site is required, as in autogenous bone grafts [23–27].

Members of the transforming growth factor beta superfamily have superior osteoinductive capabilities, with rhBMP-2 and rhBMP-7 being the most extensively studied for the treatment of bone defects. Studies have shown that rhBMP-2 can stimulate osteoblastic differentiation of mesenchymal stem cells, resulting in newly formed bone that has the same composition as that of natural bone [28, 29]. Therefore, rhBMP-2 is one of the most promising growth factors for the regeneration of osseous defects. In addition, it also overcomes most of the problems associated with both autogenous and allogenous bone grafts.

Moghadam et al. reported in 2001 the first application of rhBMP-2 in a human mandibular reconstruction. In this study, the authors demonstrated successful bone formation in a 6 cm long mandibular discontinuity defect after segmental resection, with radiographic and histological evidence of new bone formation [30]. Since then, several reports have mentioned the off-label use of rhBMP-2 in mandibular reconstruction [31–34]. For instance, Sheikh et al. reported in 2015 that a predictable reconstruction of continuity bone defects larger than 6 cm could be achieved with the use of rhBMP-2 (12 mg) and BMAC (10 ml), as an alternative to autogenous bone grafting [35].

Studies in animals and humans have shown that when rhBMP-2 is combined with absorbable collagen sponges, it can induce new bone formation even in defects of critical size [34, 36]. Absorbable collagen sponges are ideal carriers for rhBMP-2, as they strongly bind it and provide a continuous release of the protein into the surrounding milieu over a period of three weeks. Nevertheless, absorbable collagen sponges lack structural stability and have a limited ability to prevent soft tissue compression at the surgical site; this further negatively affects vascular growth in bone [35, 37].

Degidi et al. reported in 2003 that titanium mesh has the necessary stiffness for maintaining the space required for bone regeneration, especially in large mandibular or maxillary defects. Furthermore, it can be adapted and contoured into the desired form [38]. Carter et al. have reported in 2008 that one of the possible factors contributing to graft failure in cases of mandibular defect reconstruction was the lack of a space maintainer [39]. An additional benefit of titanium mesh is that it retains the graft material in position with more stability; this was especially useful, as our patient had previously undergone marginal resection [40]. The titanium mesh was easy to handle, molded around the reconstruction plate, and produced successful outcomes with neither wound dehiscence nor infections.

The rationale behind the use of BMAC is that it contains a considerable number of mesenchymal stem cells, which can differentiate into osteoprogenitor cells [41]. Bone marrow aspirate can be easily harvested by aspirating the iliac bone; the procedure is cost-effective and has low morbidity [42]. The aspirate is then concentrated by centrifugation to increase the ratio of mesenchymal stem cells to growth factors [43].

When bone marrow aspirate is combined with graft material, bone regeneration is enhanced; in some cases, it has been shown to be comparable to the results obtained from using an autograft alone. For instance, Taghavi et al. have shown in 2010 that a combination of bone marrow aspirate with allograft is a viable alternative to autogenous bone grafts [44]. Moreover, Hernigou et al. in 2014 studied approximately 1000 patients who were treated with bone grafts for delayed-union or nonunion fractures, and found that the number of adverse events was significantly lower for the bone marrow aspirate group when compared to the autograft group [45]. Furthermore, Carter et al. reported in 2008 that when rhBMP-2 was combined with bone marrow cells, two out of three cases showed successful regeneration of the mandibular defect, while one case failed to form bone due to chronic infection and lack of space maintenance by the graft [39].

The case described in this study shows that it is possible to perform mandibular reconstruction using rhBMP-2 in combination with BMAC mixed with an allograft; it is logical to combine them all with a titanium mesh to bring about bone neoformation. Even if the use of this combination seems to result in a predictable outcome, further studies are needed to determine the ideal combination of grafting materials that would result in maximal clinical and cost-effectiveness.
